# The journey so far…

**DOI:** 10.4103/0972-0707.73373

**Published:** 2010

**Authors:** Velayutham Gopikrishna

**Affiliations:** Editor-in-Chief, Department of Conservative Dentistry and Endodontics, Thai Moogambigai Dental College, Chennai, India. E-mail: hi_gopikrishna@hotmail.com

The *Journal of Conservative Dentistry*, which is the official journal of the Federation of Conservative Dentistry of India, was founded in 1998 to serve as a portal for dissemination of knowledge in the field of conservative dentistry and endodontics in India.

The journey of our journal began under the guidance and leadership of Dr. Vimal Sikri who brought out the first seven volumes of the journal and was the Editor-in-Chief from 1998 till 2004. Dr. D. Kandaswamy took over the reins as the next Editor-in-Chief from 2005 to 2008, and the journal grew immensely in scope and content during his tenure. I have been the Editor-in-Chief since 2008 till date and was entrusted with the responsibility of continuing the good work started by both these stalwarts. I started my journey with the following twin objectives:[1]

- To make our journal online and have an official journal website at par with any other international journal
- To make our journal indexed with PubMed

Medknow Publications played a very supportive role in our growth and with their guidance, we hosted our journal online at www.jcd.org.in. The submission, reviewing, and tracking of manuscript submissions were made into a completely online process in 2008. This helped in making the long-drawn reviewing process into a simple, efficient, and totally paper less procedure with a faster decision time.[1]

I would like to place on record the excellent support and help given to me by my Associate Editors, Dr. Krithika Datta and Dr. Nandini Suresh. The role played by our eminent Editorial Board and Reviewer Panel is commendable and is instrumental in improving the academic output of our journal.

The growth of our journal has been momentous and truly commendable which can be gauged by the *sixfold* increase in the article submission rate in the past 3 years [Figure 1]. This can be attributed to the growing awareness of the presence and quality of our journal which got indexed with PubMed[2];. In order to accommodate this spurt in submissions, we have increased the number of print pages as well as the number of articles being published [Figure 2]. We have also witnessed a steady growth in the number of institutional subscribers of our journal reflecting the role played by our journal as a benchmark for research in our specialty in India.

**Figure 1:**
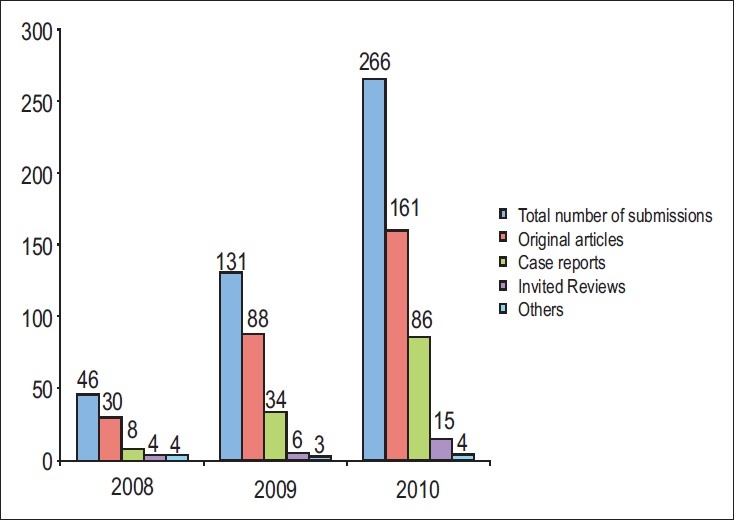
Total number of submissions along with article types for the past 3 years

**Figure 2:**
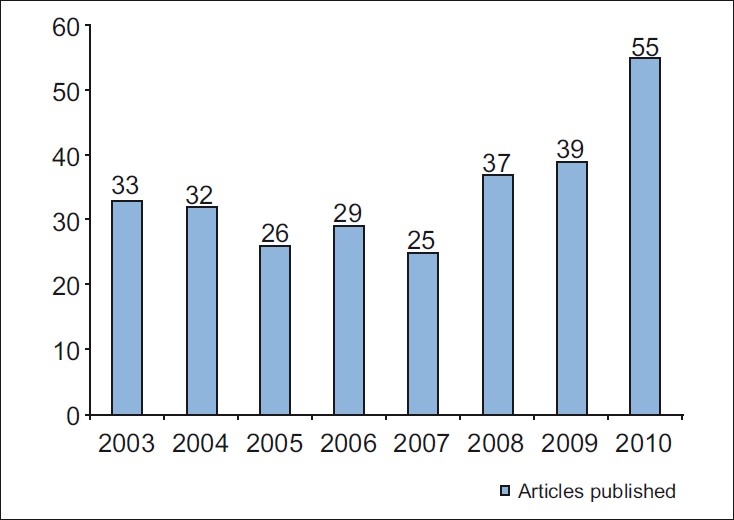
Total number of articles being published in *Journal of Conservative Dentistry* from 2003 to 2010

The 25^th^ National Conference of Federation of Operative Dentistry of India (FODI) is being held at Chennai in December 2010. On this occasion; it gives me great pleasure in bringing out this special issue to commemorate the silver jubilee celebrations of FODI. This silver jubilee issue comprises of invited review articles on important clinical and exam-oriented topics. The invited authors include the previous editors of our journal along with the leading academicians from across the country. We have also added a research article, which highlights the contribution of Indian authors to the world endodontic and conservative dentistry literature in the past two decades.

## THE JOURNEY AHEAD…

- We have already started getting article submissions from countries such as Brazil, Turkey, Iran, Malaysia, Pakistan, Japan, Senegal, Syria, and Poland. Our efforts would be to make *Journal of Conservative Dentistry*, a globally recognized journal with an international content and reach.
- We will strive to make *J. Conserv. Dent*. the premier resource journal of our specialty in Asia by 2015.
- As an immediate measure, we will increase the number of pages of our journal to 100 pages per issue in order to accommodate at least 25 articles per issue and to have an average of more than 100 article publications per year from 2011 onwards.

Looking forward for your support and inputs in making this journal an enriching academic fountainhead…

## References

[1] Gopikrishna V (2008). We have a dream…In pursuit of excellence. J Conserv Dent.

[2] Gopikrishna V, Datta K, Nandini S (2010). Journal of Conservative Dentistry is now PUBMED indexed. J Conserv Dent.

